# Pathology of Dental Caries

**Published:** 1887-07

**Authors:** E. Parsons


ARTICLE IV.

### PATHOLOGY OF DENTAL CARIES.

BY E. PARSONS, D. D. S.

[Read before the Georgia Dental Society, May, 1887.]

DEAR BRETHREN:—Your Worthy President has assigned to me the subject of Pathology of Dental Caries,—a subject which has been discussed at the meetings of nearly, if not all the dental societies in this country. The chemist and microscopist have done their utmost to reveal the cause of dental caries, and so far as I know, without adding any great amount of knowledge on the subject. There are two forces in nature's action and reaction. When these forces are harmonious they both work for our good. All creation is an out-birth fiom centre to circumstances. This is caused by the infinite source of all motion, without which physical existence is impossible. It is only by the aid of science we can understand anything of natural phenomena as to its real cause. To the uneducated, day and night is caused by the sun moving around the earth, which is only the appearance and very far from the true cause.

Human teeth are evidently designed by our All-wise Creator for an important use while we remain in this world. If they fail to accomplish the desired end we must look for the cause in imperfect organization or the activity of some destructive agent acting on them. You all know there is a great difference in the density of human teeth, hereditary, or from a want of insufficient supply of phosphate and carbonate of lime for the growth and maintenance of good, hard teeth.

It is reported by some observers that a cow while bearing and suckling, if fed on swill slops, will not only lose her teeth, but the calf will have soft teeth. If not demonstrable it is reasonable to suppose that a full supply, or a deficiency in our food and drink, is the main cause in the different density of human teeth.

Nearly all the habits of civilized man are artificial, and the laws of health are being constantly violated, hence, disease in some form or other, apparently originating from vitiated secretions, is the cause.

The pathology of dental caries opens to us a wide field for our investigation. The chemical composition of enamel and dentine are well known. Alkali may dissolve the soft tissues, but some form of animated substance, of which oxygen is the base, can alone disintegrate the hard tissues. My experiments and careful observations lead me to conclude that there are three sources of acidulated substances that cause caries in human teeth; acred secretions of the glands, acred exhalations from the lungs, and fermentation. If but one of these conditions is present, the progress is slow, but if all three are acting the progress is rapid, especially in soft teeth.

A few attribute a very different cause for the destructive work known as dental caries. The microscope reveals the fact that the microbe, a small species of animalculæ, is in vicid secretions and the debris found in decayed teeth, and hence, they conclude they are the cause of the disease. I have yet to learn that any living organisms has any power to bore a hole in the enamel of a tooth, and to use the theory is as absurd as to assert that the sun revolves around the earth, causing day and night, and is quite as far from the truth. We often find caries on the labial surface of the front incisors. What but chemical action can account for this fact? To comment on the various causes of vitiated secretions and acidulated breath, would take up too much room in this paper. I will only say they are mainly hereditary, predisposition, pernicious habits of living, and fermentation.

I cannot present anything new on the subject you have assigned me. If our people knew and would obey the laws of health, we should soon have a much healthier population in every respect, But this will not be in our day, for all bow to the goddess Fashion, and suffer the consequences. If anything new can be evolved by a discussion of the subject, I shall be glad to know it. The most intelligent and best men in our profession may be led to err as to the true cause of disease, by appearance which are often deceitful.

All true science is based on facts; all else is mere speculation. So far as we increase our knowledge of facts, we are in the true path of scientific progress, increasing our ability to easily diagnose any case that present itself for treatment.

If I am not able to attend your annual meetings, my interest in your success is the same as when with you. Considering our feeble beginning, the Georgia State Dental Society has accomplished an immense amount of good, both to our profession and the public. We have effectually closed the doors against ignorant pretenders, and elevated the status of dental science in this State. You have caused to be enacted the best laws for the protection of our profession that now exist in any State in this great republic. Every qualified dentist in this State ought to be proud of what this Society has done for them, and should, if practicable, attend our annual meetings; it is a duty they owe this Society. There is no limit to scientific research. There is now much that is not known, but is gradually being evolved, that will benefit both our profession and suffering humanity. With a united and earnest effort to make this session more useful than any previous one, you are bound to succeed. To the younger members let me say, success largely depends on earnest efforts.—*Southern Dental Journal.*

